# Editorial: Immune cell metabolism beyond energy supply – an emerging era to showcase novel roles in immune effector functions

**DOI:** 10.3389/fimmu.2026.1869468

**Published:** 2026-05-20

**Authors:** Hao Wu, Henry Kurniawan, Bin Zheng, Wei He

**Affiliations:** 1 Würzburg Institute of Systems Immunology, Max Planck Research Group, Julius-Maximilians University of Würzburg, Würzburg, Germany; 2 Luxembourg Centre for Systems Biomedicine, University of Luxembourg, Esch-Belval, Luxembourg; 3 Department of Biomedical Sciences, Cedars-Sinai Medical Center, Cedars-Sinai Cancer Institute, Los Angeles, CA, United States; 4 Department for Bioinformatics and Biochemistry, Braunschweig Integrated Centre of Systems Biology (BRICS), Technische Universität Braunschweig, Braunschweig, Germany

**Keywords:** cell metabolism, choline, fumarate, itaconate, macrophage, mesaconate, neutrophil, T cell

Over the past two decades, the field of immunometabolism has undergone a conceptual transformation – initially regarded as solely bioenergetic and biosynthetic, cellular metabolism is now recognized as a central regulator of immune cell fate, differentiation, and effector function (1). This Research Topic was conceived to highlight these non-canonical roles of metabolism, including the signaling, transcriptional, and epigenetic functions of metabolic pathways, metabolites, and enzymes.

A recurring theme across the included studies is the functional versatility of metabolic intermediates as immune regulators. Burczyk and Kolaczkowska provide mechanistic insights into how dimethyl fumarate (DMF), a derivative of the tricarboxylic acid (TCA) cycle metabolite fumarate, acts as a potent inhibitor of neutrophil extracellular traps (NETs). Utilizing an LPS-driven model, the authors demonstrate that DMF suppresses PAD4-dependent NET formation by activating the NRF2 pathway and promoting the secretion of the pro-resolving protein Annexin A1. This study positions DMF and the modulation of mitochondrial dynamics as promising pharmacological strategies for controlling excessive neutrophil activation in severe inflammatory conditions. Future intriguing questions would implicate the endogenous fumarate – does it possess the same effects as its derivative DMF, and is the dynamic level of fumarate during inflammation associated with NET activation? Similarly, Ohm et al. extend the functional repertoire of immunometabolites by demonstrating that itaconate and mesaconate exert protective effects in influenza-associated neuroinflammation. While the TCA cycle-derived itaconate has been increasingly recognized as an immunomodulatory metabolite in macrophages (2), this study advances the field by situating itaconate and its downstream metabolite mesaconate within a neuroimmune context. The authors show that these metabolites attenuate microglia-driven synaptic pathology, highlighting their therapeutic potential in neurotropic IAV infection. Despite the structural similarity of the two metabolites, phenotypic distinction between administration of itaconate and mesaconate might point to future research direction on mechanistic differences between them, such as their disparity on inhibiting succinate dehydrogenase and OXPHOS (3). In addition, cellular source and compartmentalization of itaconate and mesaconate during neurotropic infection might trigger further investigations - whether their protective effects are primarily mediated by microglia, infiltrating monocytes, or peripheral myeloid cells.

Another key insight emerging from this Topic is the tight coupling between lipid metabolic programs and immune cell phenotypes. Bouraoui et al. report that elevated expression of the fatty acid transporter CD36 and the metabolic regulator CD147 in clear cell renal cell carcinoma (ccRCC) is associated with an immunosuppressive macrophage phenotype, alongside a trend toward reduced CD8+ T cell infiltration. This observation aligns with broader evidence that lipid metabolism is a critical determinant of M2-like macrophage polarization (4), and in particular showcases the lipid-driven immunosuppression in lipid-rich tumors, of which ccRCC is a prominent representative (5). An interesting direction would be the metabolic interplay between tumor cells and these macrophages – how exactly tumor cells-derived lipids shape macrophage behaviors. Complementing this, Peesari and McAleer investigate the regulatory role of lipid metabolism in Th9 cells – a subset pivotal for both allergic responses and anti-tumor immunity. Unlike earlier work regarding glycolysis as a dominant driver of Th9 differentiation (6), this study reveals fatty acid metabolism as a previously underappreciated regulatory axis. Specifically, this study highlights PPAR-γ-driven lipid uptake and storage, alongside ACC1-driven fatty acid synthesis within Th9 cells, indicating strategic modulation of fatty acid metabolism may offer a novel therapeutic window for managing Th9-mediated disorders. Future investigation will be essential to validate these findings *in vivo*, particularly in disease models where IL-9 plays critical roles.

The importance of metabolic heterogeneity across tissue environments is emphasized by Woods and Mutlu, who describe distinct metabolic profiles of various macrophage subsets in lungs, and their metabolic dynamics in response to microenvironmental cues during pathologic conditions. The authors highlight that while tissue-resident alveolar macrophages (TR-AMs) predominantly rely on oxidative phosphorylation (OXPHOS) to maintain homeostasis in the oxygen- and lipid-rich and glucose-limited environment, monocyte-derived alveolar macrophages (Mo-AMs) and interstitial macrophages (IMs) exhibit a more glycolytically-oriented phenotype. Importantly, the review describes how environmental cues, such as the severe hypoxia and glucose influx characteristic of acute respiratory distress syndrome (ARDS), drive a HIF-1α-dependent glycolytic reprogramming in TR-AMs, which in turn governs functional adaptation of these cells. This concept is reinforced by Mandal et al., who demonstrate that high-fat diet reshapes the metabolic and inflammatory landscape in adipose tissue. Critically, rather than focusing on a single immune cell type, the authors highlight coordinated changes spanning macrophages, T cells, and dendritic cells. The link to adipogenesis further underscores the functional consequences of these immune changes, bridging immunometabolism with metabolic disease pathology. Future directions should prioritize resolving the cell-type-specific metabolic pathways driving these phenotypic changes. For instance, distinguishing the roles of glycolysis, fatty acid oxidation, and OXPHOS in different immune subsets could reveal selective intervention points.

A particularly notable contribution of this Research Topic is the re-evaluation of neutrophil metabolism, traditionally viewed as predominantly glycolytic (7). Lika et al. provide compelling evidence that mitochondrial metabolism in mature neutrophils can be rapidly reactivated upon certain stimulation, indicating that mitochondrial activity is not residual but necessary to support specific effector functions. This has also important implications for understanding how neutrophils adapt to diverse microenvironments, particularly those with varying nutrients and oxygen availability. From a therapeutic perspective, selectively targeting mitochondrial metabolism in neutrophils could offer new strategies to modulate neutrophil-dependent pathologies.

While immunometabolism has traditionally focused on glucose, amino acids, and fatty acids, Maia et al. highlight choline as a key regulator of immune cell activation, intersecting membrane biosynthesis, signaling, and epigenetic regulation. Specifically, choline serves as a precursor for phosphatidylcholine, a dominant membrane phospholipid, and as a methyl donor via its oxidation to betaine, thereby linking nutrient availability to transcriptional/epigenetic control of immune responses. This review also extends into translational relevance, discussing how alterations in choline metabolism may contribute to immune-related disorders and how choline-derived metabolites could serve as biomarkers or therapeutic targets.

**Figure 1:**
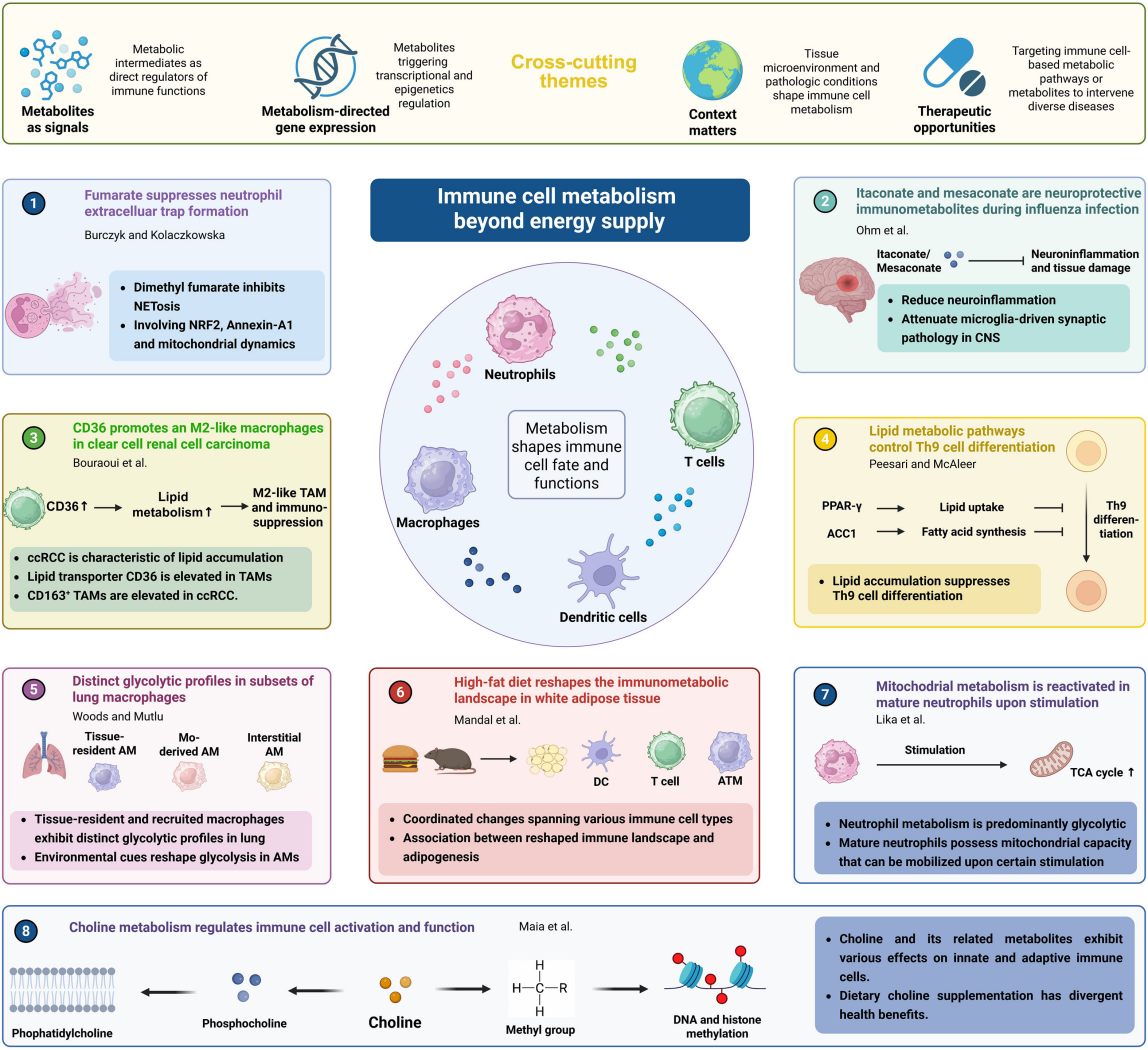
Beyond energy supply – metabolites and metabolic pathways shape immune cell fates and functions. [Created in BioRender. He, W. (2026) https://BioRender.com/oeko20z.

Collectively, the studies in this Research Topic converge on several overarching principles (**Figure 1**). First, metabolites act as signaling molecules, directly influencing immune cell behavior. Second, metabolic pathways are tightly integrated with transcriptional and epigenetic networks, enabling dynamic regulation of immune responses. Third, immunometabolism is highly context-dependent, shaped by tissue microenvironments, disease states, and systemic metabolic conditions. Finally, these insights reveal metabolism-based new therapeutic targets, allowing precise modulation of immune function in diseases.

Despite these advances, the complexity of metabolic networks, coupled with cell type-specific roles in immune responses, raises concerns regarding potential off-target effects when targeting metabolism. Moreover, the spatiotemporal dynamics of immunometabolism are not yet fully understood *in vivo*. Addressing these challenges will require the integration of multi-omics, advanced imaging, and systems biology techniques.
